# You shall not pass: how facial variability and feedback affect the detection of low-prevalence fake IDs

**DOI:** 10.1186/s41235-019-0204-1

**Published:** 2020-01-28

**Authors:** Dawn R. Weatherford, William Blake Erickson, Jasmyne Thomas, Mary E. Walker, Barret Schein

**Affiliations:** 1 Texas A&M University, San Antonio, TX USA; 2 Arkansas State University, Jonesboro, AR USA

**Keywords:** Low-prevalence effect, Facial identification, Imposter identification, Performance feedback, Receiver operating characteristic curves

## Abstract

In many real-world settings, individuals rarely present another person’s ID, which increases the likelihood that a screener will fail to detect it. Three experiments examined how within-person variability (i.e., differences between two images of the same person) and feedback may have influenced criterion shifting, thought to be one of the sources of the low-prevalence effect (LPE). Participants made identity judgments of a target face and an ID under either high, medium, or low mismatch prevalence. Feedback appeared after every trial, only error trials, or no trials. Experiment 1 used two controlled images taken on the same day. Experiment 2 used two controlled images taken at least 6 months apart. Experiment 3 used one controlled and one ambient image taken at least 1 year apart. Importantly, receiver operating characteristic curves revealed that feedback and greater within-person variability exacerbated the LPE by affecting both criterion and discriminability. These results carry implications for many real-world settings, such as border crossings and airports, where identity screening plays a major role in securing public safety.

## Significance statement

Determining an unfamiliar person’s identity is critically important to a wide variety of security-related occupations such as transportation-security screeners, border patrol agents, police officers, and other security personnel. These personnel typically compare a photo identification card (i.e., ID) to a live person before permitting access to restricted goods, services, and areas. Acceptable forms of ID are produced by a variety of agencies that embed features such as light-sensitive strips, ghost images, and material properties to help a screener distinguish a genuine from a fake ID. However, a genuine ID can still be presented by a person who is not pictured on the card. This ID is still considered fake; but, screeners need to detect the mismatched identities in order to reject it. Our research focuses on a screener’s ability to detect a fake ID under such circumstances when it is rare. We explore how response formats (e.g., yes/no decisions compared to confidence-based decisions), real-world concerns (e.g., the degree of control in manipulating within-person and between-person variability), and possible interventions (e.g., feedback) may alter the magnitude of the effect.

Unfortunately, research indicates that detecting the identity of an unfamiliar person is more difficult than it may seem (e.g., Kemp, Towell, & Pike, 1997; Robertson, Noyes, Dowsett, Jenkins, & Burton, 2016; White, Burton, Jenkins, & Kemp, 2014). Errors arise because two images of the same person can vary widely based on differences in age, hairstyles, weight, and a number of other factors. Similarly, two images of different people can look incredibly similar. Thus, determining a person’s identity requires visually searching the two different images (e.g., photo ID and live person) for two different types of cues. Observers must be able to distinguish between match cues that signal a single identity i.e., within-person variability; (e.g., Burton, 2013) and mismatch cues that signal two different identities i.e., between-person variability; e.g., (Jenkins, White, Van Montfort, & Burton, 2011).

Much like other complex visual search tasks, research shows that if one type of target—in this instance a genuine ID or fake ID—is infrequent, then an observer will often fail to identify it (Hout, Walenchok, Goldinger, & Wolfe, 2015; Rich et al., 2008; Wolfe et al., 2007). This low-prevalence effect (LPE) decreases the successful identification of weapons in real-world baggage-screening scenarios (e.g., Lau & Huang, 2010) and abnormalities during radiological screenings (e.g., Drew, Võ, & Wolfe, 2013) because both weapons and abnormalities appear less often during these searches than high-prevalence items such as aerosol cans or tumors.

Extending to work with faces, Papesh and colleagues (Papesh & Goldinger, 2014; Papesh, Heisick, & Warner, 2018) found that participants failed to detect identity mismatches when they were rare. In a series of studies, participants viewed image pairs of a target face displayed beside an ID card. For each pair, participants made untimed yes/no decisions about whether the two images represented the same person. Errors persisted on mismatch trials when mismatch prevalence was low, despite warning participants after incorrect decisions, directing participants to avoid errors through careful deliberation, and allowing participants to reconsider their initial decisions.

### The low-prevalence effect (LPE)

Although a complete explanation of the LPE is still a matter of debate, the relatively robust literature in object-identification search tasks (e.g., weapons, tumors) provides an important theoretical foundation for its origins. Studies have primarily investigated whether the LPE is driven by early visual search termination (i.e., making an identification decision before exhaustively searching an entire visual array) or criterion shifting (i.e., visually fixating upon the correct cue, but determining that it does not sufficiently exceed the threshold to be identified as such).

If these same mechanisms are applied to facial-identification tasks, the low prevalence of fake IDs can be explained as a failure to identify mismatch cues due to the wide within-person variability between IDs and the individuals presenting them. In other words, when presented with a high frequency of genuine IDs, the evidence for a mismatch decision must be sufficiently high in order to identify the ID as fake. In the absence of strong and more unambiguous visual cues that signal a mismatch (i.e., the person presenting the ID is of a different race than the photo), observers decide that two facial images belong to the same person. Following the results of their facial-identification experiments, Papesh and colleagues’ (Papesh et al., 2018; Papesh & Goldinger, 2014) findings suggest that the LPE exerts its influence by creating a context that emphasizes cue search for identity matches. Therefore, participants fail to notice the diagnostic cues that signal between-person variability on mismatch trials because they terminated their search too quickly and/or attended only to match cues. These different search strategies resulted in shorter reaction times on inaccurate mismatch trials. However, these initial investigations into how the LPE affects facial identification are limited in ways that the current studies aim to explore.

### The current research

The current studies contribute to this important area by more closely investigating factors that influence real-world security personnel and may affect criterion shifting in a serial decision-making task. First, within-person variability can affect the degree to which an individual resembles themselves over a lapse of time. Because ID photos can be valid for up to 10 years (e.g., United States passport documents), a wide variety of facial changes likely reduce the ability to adequately differentiate between an imposter presenting someone else’s ID and a legitimate person who has just changed substantially since their image was taken. In the current studies, we more strongly account for the degree to which image pairs look similar by representing different degrees of within-person variability. As a starting point, Experiment 1 used two controlled images that were taken on the same day with different cameras. To increase realism, Experiment 2 used two controlled images taken at least 6 months apart. Finally, Experiment 3 approximated the most realistic within-person variability by using one controlled image and one ambient image taken at least 1 year apart. Attention was also paid to ensuring sufficient between-person variability to approximate real-world settings, where an imposter presenting someone else’s ID must be at least adequately convincing to be believable. To represent convincing degrees of between-person variability, we created high-similarity mismatches (described in “Materials”) by pairing identities rated by an independent group of participants.

Second, feedback may influence the degree of the LPE in this face-matching task. Performance feedback in real-world security settings is delivered in a variety of ways. For instance, a screener might receive feedback by way of external validation (e.g., a screened individual is able to produce alternative forms of ID when prompted) or external information (e.g., a supervisor or confederate completes a random screening check for quality control). Although decision feedback is relatively rare compared to the vast majority of decisions that receive no additional scrutiny, it remains important to explore as a straightforward and plausible intervention strategy aimed at affecting criterion shifting. Further, professional identity screeners very typically receive feedback during their initial training period (Towler et al., 2019). Predictions about feedback are mixed, with some evidence suggesting its use as effective (Alenezi, Bindemann, Fysh, & Johnston, 2015; White, Kemp, Jenkins, & Burton, 2014) and others suggesting its use as ineffective or even detrimental (Papesh et al., 2018; Wolfe et al., 2007). Therefore, we again approached Experiment 1 as a means to replicate previous findings by providing feedback only in the case of errors (Papesh & Goldinger, 2014). Afterwards, Experiments 2 and 3 manipulated feedback more fully.

In order to consider the influences of these real-world factors, all three experiments adopted a variant of the traditional paradigm adapted from (Papesh & Goldinger, 2014) wherein participants made several decisions about whether a target face matched an ID. However, instead of yes/no judgments, participants made identity decisions on a 1–6 scale that allowed us to build receiver operating characteristic (ROC) curves (described in “Results”) and calculate discriminability and criterion. We predicted that, if the LPE exerts its influence, then both discrimination and criterion would be affected under low mismatch prevalence. However, the LPE may be reduced or nearly eliminated when image pairs represent low within-person variability (Experiment 1) compared to higher within-person variability (Experiment 2 and Experiment 3). In terms of feedback, we remained agnostic, as some evidence (Alenezi & Bindemann, 2013; White, Kemp, et al., 2014) would predict that feedback will increase discriminability and criterion (i.e., combat criterion shifts and decrease the likelihood of early search termination), whereas other evidence (e.g., Papesh et al., 2018) would predict that feedback will decrease discriminability and criterion (as a function of drawing attention to the low mismatch prevalence, thereby exacerbating the effect).

## Experiment 1

### Method

#### Participants

Undergraduate students (*N* = 91; *M*_age_ = 19 years; 68 female) participated in the experiment in exchange for partial course credit. Power analyses confirmed the sufficiency of this sample size for all omnibus tests (i.e., *β* − 1 > .95). Self-reported race reflected a diverse sample (15 Black/African American, 70 White/Caucasian, 1 Hispanic/Latino, 4 Asian/Asian-American/Pacific Islander, and 1). All participants reported normal or corrected-to-normal vision.

#### Materials

**Fig. 1:**
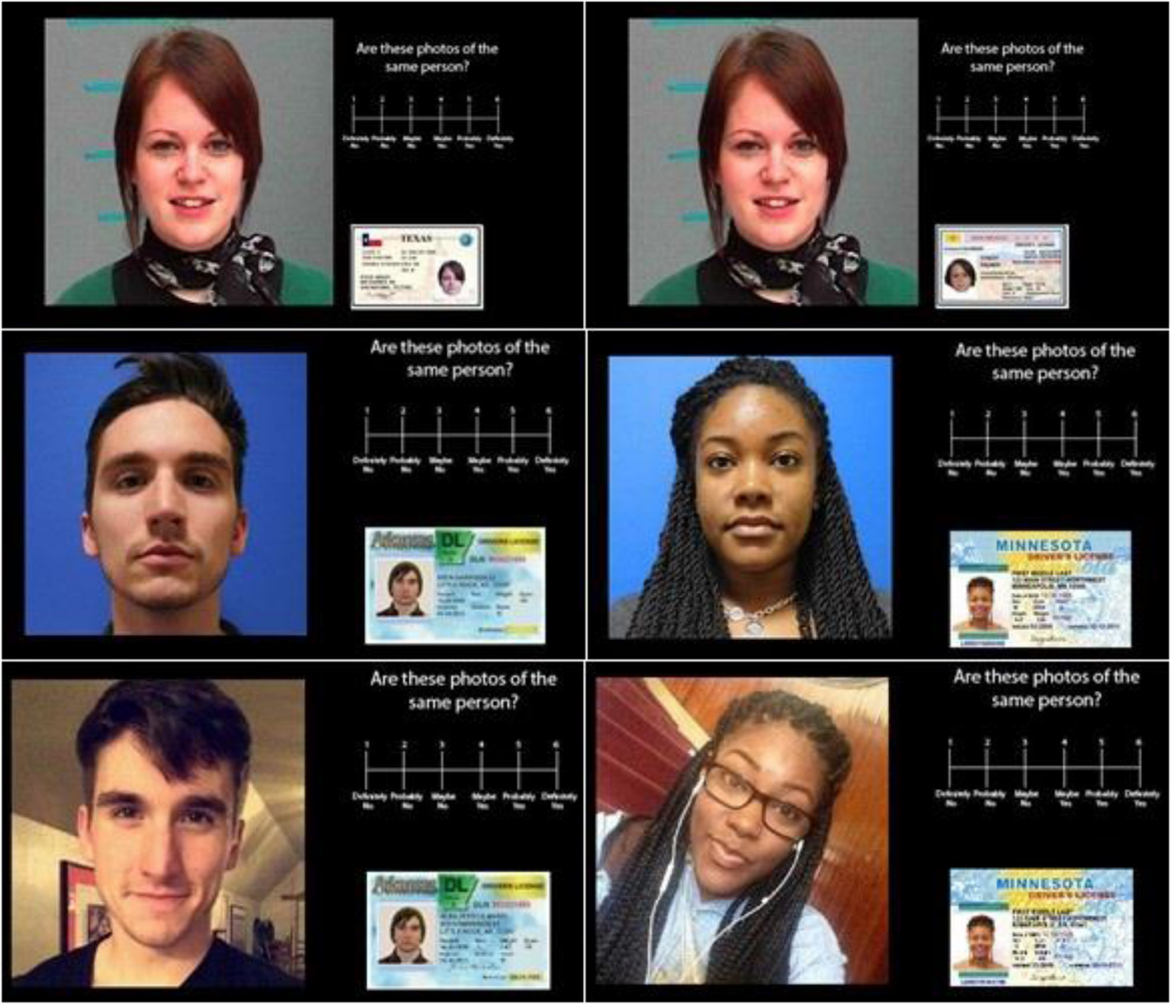
Example stimuli of identities used in Experiment 1 (top row), Experiment 2 (middle row), and Experiment 3 (bottom row). The left column represents match pairs, whereas the right column represents mismatch pairs. All images used with permission

One-hundred and forty image pairs were selected for use in the experiment. Each match pair contained two different front-facing photographs of the same person, taken on the same day with two different cameras (Glasgow Unfamiliar Face Database, http://www.facevar.com/downloads). Adapting Papesh and Goldinger (2014), each trial presented a target face (approximately 5 in. by 5 in.) beside an ID card (approximately 2.25 in. by 1.5 in.; see Fig. 1). EPrime presented images to participants on a 22-inch monitor such that target identities the occupied the larger portion of the left side of the screen and ID card identities were embedded within one of several prototypical ID card images on the right side of the screen.

Mismatch identity pairs displayed two photographs of two different people (see Fig. 1, right column). Mismatch identities were paired using reported similarity ratings (all between 0.3 and 0.6 (M = 0.40, SD = .09), see Bruce et al., 1999; Burton, White, & McNeill, 2010)^1^ Match and mismatch identities were fully counterbalanced and no images repeated across trials, such that each identity was equally likely to appear beside another photograph of themselves as they were a photograph of another person.

#### Design and procedure

After providing informed consent, participants made 140 untimed identity decisions under either high (80%), medium (50%), or low (20%) mismatch prevalence. Participants answered “Are these images of the same person?” by selecting a number on a 1–6 scale (1 = *definitely no*, 6 = *definitely yes*). To replicate the experimental conditions of Papesh and Goldinger (2014), participants viewed a 2-s penalty screen following incorrect decisions on match trials and a 4-s penalty screen following incorrect decisions to mismatch trials.^2^ After completing all trials, participants provided demographic information and were debriefed.

### Results

To allow more direct comparison with findings derived from yes/no judgments in previous studies, we first calculated accuracy by collapsing the response scale, with responses 1–3 coded as correct for mismatch trials and responses 4–6 coded as correct for match trials. These collapsed values were used to calculate accuracy and signal detection analyses. After satisfying that connection with the literature, we considered the full range of responses to construct ROC curves that more completely explore discriminability across all levels of confidence.

#### Accuracy

**Fig. 2:**
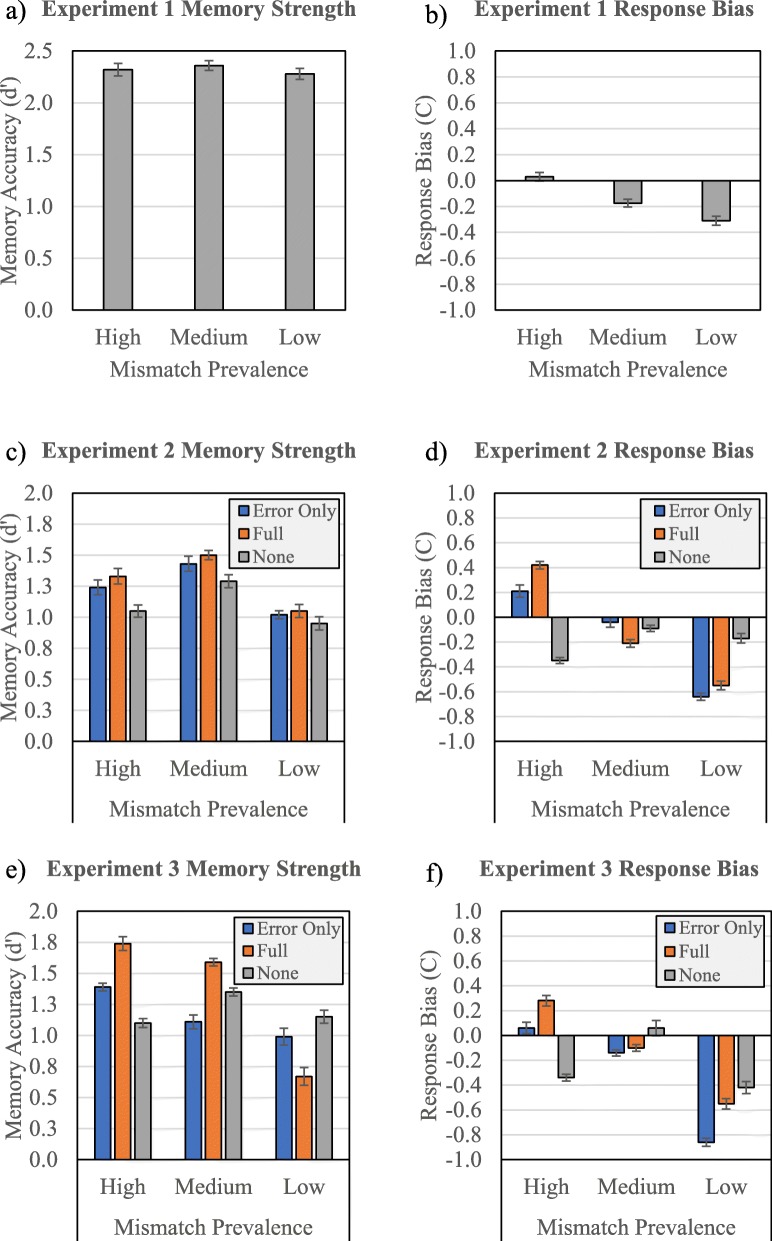
Signal detection measures of discriminability (*d’*) and response bias (*C*) at each mismatch prevalence rate (high, medium, and low) for Experiments 1 (**a** and **b**), 2, (**c** and **d**), and 3 (**e** and **f**). Figures for Experiments 2 and 3 additionally break down data by feedback condition (error-only, full, and none)

We analyzed accuracy using a 2 (Match Type-within: match, mismatch) × 3 (Mismatch prevalence-between: 80%, 50%, 20%) mixed-methods analysis of variance (ANOVA). Unless otherwise stated, we set alpha at .05 and corrected for Type 1 error inflation across all statistical tests using Bonferroni post-hoc analyses. We found a main effect of match type, *F* (1,88) = 17.768, *p* < .001, *η*^2^_*p*_ = .168 and no main effect of prevalence, *F* (2,88) = .99, *p* = .375, *η*^2^_*p*_ = .022. However, this main effect was qualified by an interaction between match type and mismatch prevalence, *F* (2,88) = 7.33, *p* = .001, *η*^2^_*p*_ = .143. As can be seen in Fig. 2a, planned follow-up analyses indicated that participants were more accurate for match trials (*M =* .89, *SD* = .09) than mismatch trials (*M* = .82, *SD =* .11) across all mismatch prevalence conditions (a trend we address when considering the influence of facial variability below); however, accuracy followed a mirror effect across different prevalence rates. Error rate analysis followed the same pattern and magnitude of results.

#### Signal detection measures

We also analyzed performance using signal detection measures, sensitivity (*d*’):

1 $$ {d}^{\prime }=z\left( False\ alarms\right)-z(Hits) $$

where higher values of *d’* indicate superior recognition memory while accounting for response bias. To account for extreme performance levels (e.g., hit or false alarm rates of zero), extreme values are replaced by 1–2/*N* for rates of 1 or 2/*N* of 0, where *N* represents the number of trials of that type. We were also calculated response criterion (*C*);

2 $$ C=\frac{-1\left(\left( False\ alarms\right)+z(Hits)\right)}{2} $$

Figure 1 displays all data by prevalence and feedback conditions. For *d’*, a between-subjects ANOVA revealed no main effect of mismatch prevalence, *F* (2,88) = .19, *p* = .831, *η*^2^_*p*_ = .004. For *C*, a between-subjects ANOVA revealed a main effect of mismatch prevalence, *F* (2,74) = 9.89, *p* < .001, *η*^2^_*p*_ = .194. As predicted by the criterion-shift explanation of the LPE, mismatch prevalence affected criterion in linear fashion, with the low mismatch prevalence group demonstrating a more liberal criterion than the high- and medium-prevalence conditions.

#### Area under the curve

**Fig. 3:**
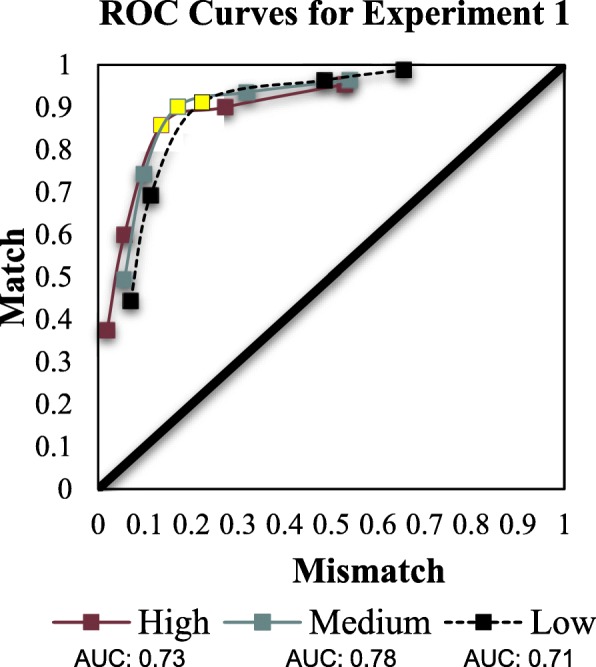
Receiver operating characteristic (ROC) curves by mismatch prevalence group. Area under the curve (AUC) calculated using the trapezoidal method. To ease comparison, the third point on each ROC for high, medium, and low mismatch prevalence is highlighted to show a slight rightward shift along the x-axis

A further analysis that can illuminate the effect of mismatch prevalence rates on criterion shifting simultaneously considers discriminability across a range of criterion values. To this end, we calculated area under the curve (AUC). The cumulative proportions of “1”,“2”, “3”, “4”, “5”, and “6” responses made by each participant within each prevalence condition from 6 (the highest criterion level) to 2 (the lowest criterion level) were calculated for each pair type (match or mismatch) and plotted in ROC space for three curves. The space is arranged such that match proportional accuracy is plotted along the vertical axis from 0 at the origin to 1 at its maximum for match decisions, and along the horizontal axis from 0 at the origin to 1 at its maximum for mismatch decisions. Thus, a diagonal from coordinate 0,0 to 1,1 indicates chance performance. Coordinates above this diagonal indicate accuracy above chance; coordinates below this diagonal indicate accuracy below chance. As can be seen from Fig. 3, accuracy was generally high in each mismatch prevalence condition as indicated by each curve bowing toward the upper left of the ROC space.

Next, we computed the partial area under the curve (pAUC) scores for each ROC curve and conducted pairwise comparisons among each mismatch prevalence condition (see Table 1). Scores for full AUCs typically range from .50 (chance performance) to 1.00 (perfect performance). Although several methods of calculating these scores exist, most involve extrapolating the left-most and right-most data points of each ROC curve to the 0,0 and 1,1 coordinates on the plot. This approach puts in jeopardy interpretations made by comparing two ROCs on a plot which do not perfectly overlap along the x-axis, as different degrees of extrapolation are needed for each. Therefore, for each ROC curve comparison, we compared only those portions where the two curves do overlap. We used the pROC toolbox (Robin et al., 2011) in R to compute pAUC scores for the curves corresponding to each prevalence level within sub-portions of the aggregate ROCs that overlapped along the x-axis of the ROC plots. In addition, because there were three comparisons in total, the alpha level for significance decisions was adjusted to .017.

**Table 1:** Lower and upper receiver operating characteristic (ROC) curve overlap boundaries used for each partial area under the curve (pAUC) analysis, including *D* value for each comparison

Mismatch prevalence comparisons	Partial ROC boundaries		*D*
	Lower	Upper	
Experiment 1			
Low prevalence vs. medium prevalence	.44	.94	5.86a
Low prevalence vs. high prevalence	.44	.93	2.72a
Medium prevalence vs. high prevalence	.44	.93	3.33^a^
Experiment 2			
Low prevalence vs. medium prevalence	.29	.92	4.21^a^
Low prevalence vs. high prevalence	.41	.92	2.38^a^
Medium prevalence vs. high prevalence	.41	.96	1.20
Experiment 3			
Low prevalence vs. medium prevalence	.31	.90	2.67^a^
Low prevalence vs. high prevalence	.39	.90	2.73^a^
Medium prevalence vs. high prevalence	.39	.96	.77

*Note:* ^a^Indicates a significant difference at *p* < .017, corrected for multiple comparisons

For the comparison between low mismatch prevalence and medium mismatch prevalence, the area spanned by the low condition (pAUC = .44) was less than the area spanned by the medium condition (AUC = .47), *D* = − 5.85, *p* < .001. For the medium and high conditions, the area spanned by medium mismatch prevalence (pAUC = .45) was greater than high mismatch prevalence (pAUC = .44), *D* = 3.47, *p* < .001. Comparisons between the low and high conditions yielded no differences in pAUCs.

### Discussion

Experiment 1 confirmed our initial predictions and replicated the findings of Papesh and Goldinger (2014) that low mismatch prevalence decreased accuracy when we collapsed across the range of 1–6 judgments to replicate the yes/no paradigms that were previously adopted (e.g., Papesh et al., 2018; Papesh & Goldinger, 2014). Data replicated the classic mirror effect across the three mismatch prevalence rates, with match accuracy performance *increasing* from high to low mismatch prevalence and mismatch accuracy *decreasing* from high to low mismatch prevalence. Next, we explored criterion and discriminability using ROC curves. This measure, previously unavailable with the yes/no format of other work, confirmed that prevalence rates affected criterion. However, this more sensitive instrument revealed a much smaller difference on discriminability by prevalence — participants’ overall discriminability was very high, regardless of mismatch prevalence condition.

The results of the ROC curves are promising for translation to real-world identification screening tasks. However, some marked differences between our design and the conditions of real-world security scenarios are worthy of consideration before making any claims about generalizability. Therefore, Experiments 2 and 3 examined the magnitude of the LPE across three additional differences that should theoretically affect criterion.

## Experiment 2

### The influence of facial variability

Real-life identification tasks rarely involve comparing a photo ID to a person when that ID was taken on the same day. Further, identity screeners see people from all over the world who do not share such a high degree of visual similarity. Subsequently, many identity-matching studies underscore the need to control for both within-person and between-person variability in a way that more strongly maps onto these real-world conditions (Burton, 2013; Megreya, Sandford, & Burton, 2013). Although constraining experimental materials might increase internal validity, it also might produce outcomes that drastically underestimate externally valid facial variability and, therefore, mask potential generalizability.

The Glasgow Unfamiliar Face Database (GUFD) (Experiment 1) includes images taken on the same day, which limits within-person variability, of mostly (if not exclusively) young, light-skinned individuals, which limits between-person variability. This database’s limited facial variability likely affected participants’ criteria based upon information at the item-level (i.e., considering only information about the presented image pair on screen) and series-level (i.e., considering information across successive trials). At the item-level, low within-person variability may have contributed to the significantly higher accuracy for our match than mismatch trials overall. It stands to reason that images taken approximately 15 min apart would bear a striking resemblance to one another in terms of a variety of both noticeable (e.g., hairstyle) and subtle (e.g., skin luminance) visual cues. Therefore, the low degree of within-person variability increases matched cues across images. At the series-level, such unrealistically high similarity between match pairs likely made mismatch cues more distinctive in contrast i.e., more obvious difference in the context of similarity; (e.g., Hunt, 2006). Mismatches may have popped out more so than would have been expected in a more variable image set, and, therefore, reduced participants’ tolerance for perceptual differences as they calibrated their expectations for natural variations in a person’s appearance from day to day (see also, Menon, White, & Kemp, 2015a for a targeted approach to manipulating expected identity variation that produced findings that align with our rationale).

Previous identity matching studies in the low-prevalence literature confirm that stimulus variability can alter the interpretation of results. For example, Bindemann, Avetisyan, and Blackwell (2010) also used the GUFD to compare identity matching performance across five different experiments. Mismatch prevalence was varied in the first 49 of 50 trials, such that participants with saw either 24 mismatches (high) or 0 mismatches (low). When the authors compared performance on a final critical mismatch trial, several variations of the study confirmed that the high mismatch prevalence group committed more mismatch errors. On the face of it, these results suggest that low mismatch prevalence (2%) did not produce the classic LPE. However, we caution against that interpretation on the basis of how mismatch trials were selected. The authors strategically selected the critical mismatch image pairs with higher similar ratings (*M =* .56) than noncritical mismatch image pairs (*M* = .20). In other words, participants in the high-prevalence group saw more obvious mismatches that increased their likelihood of missing a less obvious mismatch.

More recent work has added to our understanding of the LPE by using face databases containing images taken multiple days (if not years in some instances) apart, (Papesh and Goldinger, 2014; Papesh et al., 2018) and another recent study (Susa, Michael, Dessenberger, & Meissner, 2019) found the LPE. This latter study used images specifically designed to test cross-race influences, and, therefore, portrayed a wider-degree of within- and between-person variability. Therefore, we considered it important to utilize an image set that more strongly represents real-world facial variability. Experiments 2 and 3 included a racially diverse database with multiple images of each identity taken with different cameras at different times.

### The influence of differing prevalence rates

Although our low mismatch prevalence group did experience fewer mismatch trials overall, a 20% mismatch prevalence rate is still far greater than real-world settings. Although no exact figure exists, one could estimate that a very small percentage (i.e., < 1%) of passengers present a fake ID, making it quite a bit rarer than we have accounted for here. Nevertheless, participants in both the high and low mismatch prevalence groups were sensitive to the imbalance, which suggests that prevalence effects follow a continuous function. Put another way, participants made more errors on whichever type of trial was relatively rarer (either 20% mismatches or 20% matches). Therefore, if participants were sensitive enough to modify their decisions in response to differences between 80/20 prevalence rates, then our results likely *underestimate* the errors one could expect in a typical identity-screening scenario. Studies outside of the facial recognition literature support this interpretation by confirming that a greater degree of imbalance (Mitroff & Biggs, 2014; Wolfe & Van Wert, 2010) increases the magnitude of the LPE. Although an ultra-rare prevalence condition in this particular task would introduce its own set of problems, Experiments 2 and 3 adopted a greater degree of imbalance (i.e., 90/10 and 10/90) against which to compare to an equivalent prevalence group.

### The influence of feedback

Although feedback in real-world settings is rare, it is naturally skewed toward combating mismatch errors for two primary reasons. First, most IDs that screeners check are genuine and presented by their rightful owner (a fact also responsible for the LPE). Second, screeners in many settings (e.g., airport security, liquor store cashiers) may never be made aware of that they accepted a fake ID because they are unlikely to encounter that individual again. However, they are made aware when they erroneously reject an authentic ID if the individual is able to provide alternative means of identification. Therefore, investigating the effect of feedback is crucial when considering empirically driven training regimens designed to reduce the LPE, particularly if it persists under these real-world situations. Because the LPE literature with facial-identification tasks is both fractured in its use and findings with feedback, some overview of the efficacy of feedback interventions on learning is in order before we proceed with our specific predictions.

The position that feedback improves performance dates in psychological science to the earliest days of behaviorism (e.g., Thorndike’s (1927) *Law of Effect*). Many behaviorists eschewed theory, so the benefits of feedback were often taken at face value. Kluger and DeNisi’s (1996) Feedback Intervention Theory provides a framework from which we can make predictions relevant to security screening. Feedback Intervention Theory presupposes that five components are required for feedback to modify performance at a given task. First, a gap exists between the performance upon which feedback is given and the “standard” (i.e., the desired level of performance). Second, the various goals related to task performance are organized hierarchically. Third, feedback can only regulate future behavior when the gap between current performance and the standard receives the individual’s attention. Fourth, attention moderates the ranking that a particular standard has in the goal hierarchy. Fifth and finally, feedback interventions affect behavioral outcomes by shifting attention within this hierarchy, thereby reordering the various goals.

The success or failure of a particular feedback intervention relies on the specific aspects of the task to which feedback draws attention. Given this, Kluger and DeNisi (1996) concluded that feedback exerts its greatest influence when tasks are sufficiently challenging, yet concrete (i.e., tasks that are too easy, difficult, or nebulous are unlikely to benefit), and when it focuses attention towards cues related to the task’s standard rather than to the individual (e.g., mere praise or admonishment may alter feelings of self-efficacy, but they do not necessarily affect performance).

Central to the current studies is the differential attention paid to match and mismatch cues observable in faces presented side-by-side, and how feedback affects where these cues lie within matching task’s goal hierarchy. Both cue types are shared within and between face-identity images dichotomously (i.e., facial features can only match or mismatch). The observer, then, must decide whether match cues outweigh mismatch cues when deciding whether two face images belong to the same identity. According to Feedback Intervention Theory, feedback would operate in a face-matching task by shifting attention within the goal hierarchy to cues that are most likely to match between face images belonging to the same identity. Therefore, it would make the visual system more sensitive to within-person variability.

Indeed, multiple studies demonstrate that feedback improves unfamiliar face-matching performance (Alenezi & Bindemann, 2013; White, Kemp, et al., 2014). However, the opposite influence of feedback has also been argued (Papesh et al., 2018). To date, the facial-identification paradigms that failed to find an effect of low mismatch prevalence (Bindemann et al., 2010; Stephens, Semmler, & Sauer, 2017) did not incorporate trial-by-trial feedback. Under such conditions, Feedback Intervention Theory would predict that the absence of feedback would not draw attention to the imbalanced trial types, thus not altering the cue hierarchy. Participants may not even be explicitly aware of the different mismatch prevalence rates and assume successful task performance. To more directly test this possibility, we more fully explored feedback with our modified paradigm using image sets with high facial variability.

### Predictions

If more realistic facial variability (as would be the case with a greater lapse in time between images of a diverse group of people) also increases the difficulty of the task and exacerbates the LPE, then discriminability should decrease when mismatches are either infrequent or frequent (compared to when matches and mismatches are balanced). We may also see evidence of criterion shifting, as a greater degree of within-person variability might interact with prevalence to shift criterion even more liberally under low mismatch prevalence and conservatively under high mismatch prevalence than with more similar-looking match pairs. In contrast, if a wider degree of between-person and within-person variability does not interact with mismatch prevalence, then we expect to replicate the criterion shifting seen in Experiment 1, but not necessarily see differences in discriminability by mismatch prevalence.

With regards to our predictions about feedback, Feedback Intervention Theory would predict that varying mismatch prevalence will interact with the effect of feedback on mismatch accuracy in fairly straightforward ways: Low mismatch prevalence within a set of trials will yield fewer opportunities to make mismatch errors, and, therefore, fewer opportunities to modify the standard cue hierarchy toward attending to mismatch cues. This finding should be true, and reduce discriminability, in either of the imbalanced mismatch prevalence rates. When mismatch prevalence is low, feedback will increase the weight of match cues in the hierarchy, resulting in imbalanced performance favoring match trials (but overall reduced discriminability). When mismatch prevalence is higher, feedback will increase the weight of mismatch cues in the hierarchy, resulting in imbalanced performance favoring mismatch trials (but overall reduced discriminability).

### Method

#### Participants

Undergraduate students (*N* = 83) participated in the experiment (*M*_age_ = 24.1 years; 62 female) in exchange for partial course credit. Power analyses confirmed the sufficiency of this sample size for all omnibus tests (i.e., *β* − 1 > .88). Self-reported race reflected a diverse sample (7 Black/African American, 17 White/Caucasian, 54 Hispanic/Latino, 1 Asian/Pacific Islander, and 3 other with 1 failing to respond). All participants reported normal or corrected-to-normal vision.

#### Materials

For Experiments 2 and 3, we used a face database with a complete collection of images for each of 100 unique identities between the ages of 18 and 30 years and ethnic/racial categories aligned with the 2010 U.S. Census (*Selfies for Science*; Weatherford, Ottoson, Cocherell, & Erickson, 2016 used with permission). In order to systematically control non-face image properties, acceptable photographs were cropped to a standard size and minor artificial features (e.g., earrings) were naturalistically removed using Adobe Photoshop CS7. Front-facing static images for each identity included (1) a high-resolution image taken with a neutral expression in front a blue background, (2) a student ID photograph taken on a different day with a different camera, and (3) a participant-submitted ambient facial image (i.e., selfie) that included full face, no filters or digital alterations, and was taken at least 1 year prior to the high-resolution controlled image.^3^ To create plausible mismatch trials, identities were paired using reported similarity ratings provided by an independent group of raters. Match and mismatch identities were fully counterbalanced and no images repeated across trials.

#### Design and procedure

The experiment included a 3 (Mismatch prevalence: high 90%, medium 50%, or low 10%) × 3 (feedback: error, full, or none) between-participants factorial design. Participants made 100 untimed decisions about whether a target image (a high-resolution controlled image) represented the same person as an image embedded in an ID card (a student ID image). The procedure was identical to Experiment 1 with the exception of the feedback manipulation. In the error-only feedback condition, participants viewed penalty screens as described in Experiment 1. In the full-feedback condition, participants viewed a 2.5-s feedback screen after every trial. In the no-feedback condition, participants viewed a 2.5-s black inter-stimulus interval screen after each trial.

### Results

#### Accuracy

We analyzed our data using a 2 (Match Type-within: match, mismatch) × 3 (Mismatch prevalence-between: high, medium, low) × 3 (Feedback-between: error, full, or none) mixed-methods ANOVA. For accuracy (Fig. 3), there was a main effect of match type, *F* (1,74) = 20.35, *p* < .001, *η*^2^_*p*_ = .216, a main effect of mismatch prevalence, *F* (2,74) = 6.99, *p* = .002, *η*^2^_*p*_ = .159, but no main effect of feedback *F* (2,74) = .33, *p* = .717. However, these main effects were qualified by an three-way interaction, *F* (4,74) = 9.36, *p* < .001, *η*^2^_*p*_ = .336 (Figure available in “Additional file 1*”*). Simple main effects of mismatch prevalence on errors within each level of feedback revealed a simple main effect of mismatch prevalence within error-only feedback, *F* (2, 74) = 3.14, *p* = .049, *η*^*2*^_*p*_ = .078, and also within full feedback, *F* (2,74) = 3.39, *p* = .039, *η*^*2*^_*p*_ = .084. The no-feedback condition yielded no simple effect of mismatch prevalence.

These results followed the same pattern as observed in Experiment 1. In line with the predictions of Feedback Intervention Theory, feedback improved performance of high-prevalence trial types (e.g., high prevalence of mismatch trials or high prevalence of matched trials) in the imbalanced conditions at the expense of low mismatch prevalence trial types.

#### Signal detection measures

As with Experiment 1, we also considered the criterion-shift explanation of the LPE. Signal detection measures are represented graphically in Fig. 1. For *d’*, a between-subjects ANOVA revealed a main effect of mismatch prevalence, *F* (2,74) = 5.16, *p* = .008, *η*^2^_*p*_ = .122, but no main effect of feedback, *F* (2,77) = 1.21, *p* = .304, *η*^2^_*p*_ = .032. The interaction between feedback and mismatch prevalence did not reach significance. For *C*, a between-subjects ANOVA revealed a main effect of mismatch prevalence, *F* (2,74) = 20.98, *p* < .001, *η*^2^_*p*_ = .362, but no main effect of feedback, *F* (2,74) = .572, *p* = .567, *η*^2^_*p*_ = .015. However, any main effects were qualified by an interaction between mismatch prevalence and feedback, *F* (4,74) = 9.61, *p* < .001, *η*^2^_*p*_ = .342. Simple main effects tests of mismatch prevalence within each level of feedback revealed a simple main effect of mismatch prevalence within error-only feedback, *F* (2, 74) = 18.24, *p* < .001, *η*^2^_*p*_ = .330, and also within full feedback, *F* (2,74) = 21.90, *p* < .001, *η*^2^_*p*_ = .372. The no-feedback condition yielded no simple effect of mismatch prevalence.

#### Area under the curve

**Fig. 4:**
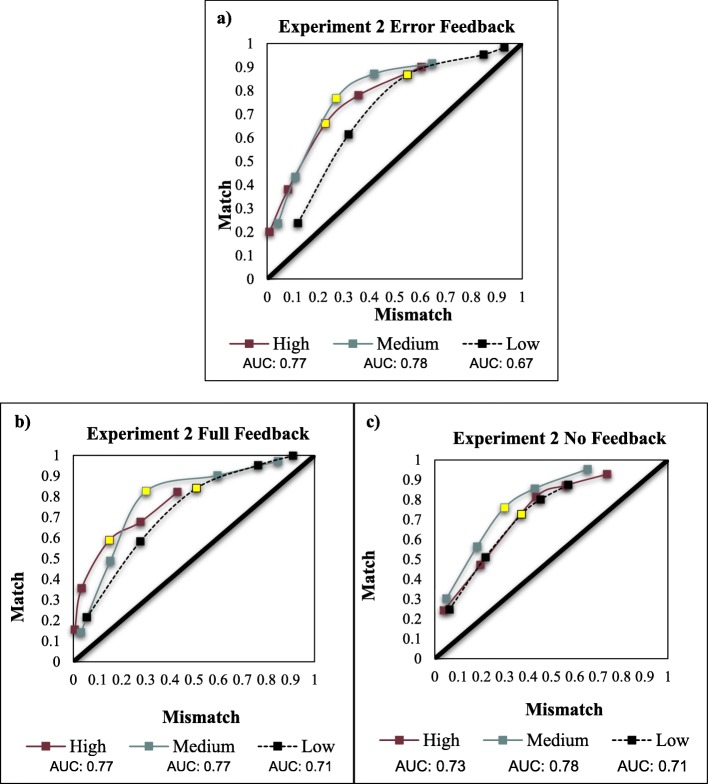
Receiver operating characteristic (ROC) curves by mismatch prevalence group for the error-only feedback (**a**), full-feedback (**b**), and no-feedback (**c**) conditions in Experiment 2. Area under the curve (AUC) calculated using the trapezoidal method. To ease comparison, the third point on each ROC for high, medium, and low mismatch prevalence is highlighted. Although the error-only and full-feedback conditions show a more robust rightward shift along the x-axis, differences in the no-feedback group were only slight

Due to the inordinately large number of comparisons in our complete design using all possible mismatch prevalence and feedback variations, we collapsed across feedback conditions to allow more straightforward comparison to Experiment 1’s results. As seen in Fig. 4, overall discriminability reduced compared to the high performance in Experiment 1. For the comparison between low mismatch prevalence and medium mismatch prevalence, the area spanned by low prevalence (pAUC = .42) was less than the pAUC for medium prevalence (pAUC = .48), *D* = 4.21, *p* < .001. For the low mismatch prevalence to high mismatch prevalence comparison, low prevalence spanned a smaller area (pAUC = .34) than high prevalence (pAUC = .39), *D* = 2.38, *p* = .006. Medium and high conditions were equivalent, *p* > .2.

### Discussion

Similar to Experiment 1, we find support for criterion shifting. Unlike Experiment 1, Experiment 2’s paradigm resulted not only in differences in criterion, but also discriminability, by mismatch prevalence. These findings align well with other recent LPE studies in the facial-identification literature (e.g., Papesh et al., 2018; Papesh & Goldinger, 2014; Susa et al., 2019) and perhaps explain the lack of an effect in others (e.g., Bindemann et al., 2010; Stephens et al., 2017). This latter study and others like it used image sets with low between-person variability (e.g., Glasgow Face Matching Test; Burton et al., 2010). By contrast, we used an image set with more realistic variability and external validity. With a greater span of time between the two comparison images, match trials were likely less strikingly obvious.

Additionally, we found that feedback interacted with prevalence to produce differences in discriminability and criterion. As Feedback Intervention Theory predicts, any imbalance in trial types shifts the ranking of cues in the hierarchy when feedback (either error or full) emphasizes it. In response to imbalanced prevalence rates, participants shifted their criterion to either be more liberal when fake IDs were rare or more conservative when they were frequent.

Having established two different effects using different stimulus sets, Experiment 3 aimed to replicate and extend results to the most visually variable image set in the series. Ambient images may be the most representative of how individuals may present themselves during identification screenings. If this increasingly greater challenge between images follows the pattern of results of Experiment 2, we can have greater assurance of patterns of behavior that might emerge in real-world settings. If, however, Experiment 3’s results look more like Experiment 1, then we might expect criterion shifting, but not reduced discriminability, by prevalence and feedback conditions. Either outcome would be informative for future research and policy recommendations.

## Experiment 3

### Methods

#### Participants

Undergraduate students (*N* = 85) participated in the experiment (*M*_age_ = 25.7 years; 67 female) in exchange for partial course credit. Power analyses confirmed the sufficiency of this sample size for all omnibus tests (i.e., *β* − 1 > .85). Self-reported race reflected a diverse sample (7 Black/African American, 10 White/Caucasian, 61 Hispanic/Latino, 3 Asian/Pacific Islander, and 3 other with 1 failing to respond). All participants reported normal or corrected-to-normal vision.

#### Materials, design, and procedure

Experiment 3 was identical to Experiment 2 in all respects except the comparison images. Participants made 100 untimed decisions about whether a target image (an ambient image) represented the same person as an image embedded in an ID card (a student ID image).

### Results

#### Accuracy

As with Experiments 1 and 2, we analyzed accuracy using a 2 (Match Type-within: match, mismatch) × 3 (Prevalence between: high, medium, low) × 3 (Feedback between: complete, error-only, none) mixed-methods ANOVA. We found a main effect of match type, *F* (1,74) = 18.74, *p* < .001, *η*^2^_*p*_ = .202, a main effect of mismatch prevalence, *F* (2,74) = 5.10, *p* = .008, *η*^2^_*p*_ = .121, but no main effect of feedback *F* (2,74) = 1.32, *p* = .273. However, these main effects were qualified by an interaction between match type, prevalence, and feedback, *F* (4,74) = 2.39, *p* = .037, *η*^2^_*p*_ = .16 (Figure available in “Additional file 1”). Simple main effects tests of mismatch prevalence on errors within each level of feedback revealed a simple main effect of mismatch prevalence within error-only feedback, *F* (2, 77) = 4.03, *p* = .022, *η*^2^_*p*_ = .095, and also within full feedback, *F* (2,77) = 10.06, *p* < .001, *η*^2^_*p*_ = .207. The no-feedback condition yielded no simple effect of mismatch prevalence.

#### Signal detection measures

We treated and analyzed signal detection measure data in the same fashion as Experiments 1 and 2. For *d’*, a between-subjects ANOVA revealed a main effect of mismatch prevalence, *F* (2,77) = 5.62, *p* = .005, *η*^2^_*p*_ = .127, but no main effect of feedback, *F* (2,77) = 2.19, *p* = .119, *η*^2^_*p*_ = .054. However, any main effects were qualified by an interaction between mismatch prevalence and feedback, *F* (4,77) = 2.62, *p* = .041, *η*^2^_*p*_ = .12. Simple main effects of mismatch prevalence on *d’* within each level of feedback revealed only a simple main effect of mismatch prevalence within the full-feedback condition, *F* (2, 77) = 9.43, *p* < .001, *η*^2^_*p*_ = .197. For *C*, a between-subjects ANOVA revealed a main effect of mismatch prevalence, *F* (2,77) = 18.14, *p* < .001, *η*^2^_*p*_ = .320, but no main effect of feedback, *F* (2,77) = 1.68, *p* = .194, *η*^2^_*p*_ = .042. However, any main effects were qualified by an interaction between mismatch prevalence and feedback, *F* (4,77) = 3.35, *p* = .014, *η*^2^_*p*_ = .148. Simple main effects of mismatch prevalence on *C* within each level of feedback revealed a simple main effect of mismatch prevalence within error-only feedback, *F* (2, 77) = 11.73, *p* < .001, *η*^2^_*p*_ = .234, within full feedback, *F* (2,77) = 9.23, *p* < .001, *η*^2^_*p*_ = .193, and also within no feedback, *F* (2, 77) = 4.64, *p* < .013, *η*^2^_*p*_ = .108.

#### Area under the curve

**Fig. 5:**
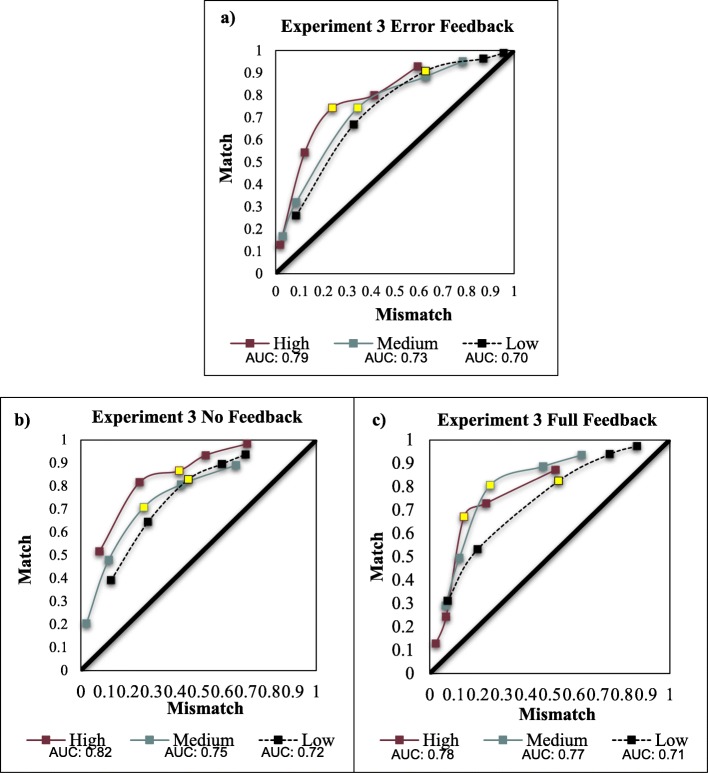
Receiver operating characteristic (ROC) curves by mismatch prevalence group for the error-only feedback (**a**), no-feedback (**b**), and full-feedback (**c**) conditions in Experiment 3. Area under the curve (AUC) calculated using the trapezoidal method. To ease comparison, the third point on each ROC for high, medium, and low mismatch prevalence is highlighted. Although the error-only and full-feedback conditions show a more robust rightward shift along the x-axis, differences in the no-feedback group were more slight, with high and low mismatch prevalence behaving similarly

Partial AUC analysis for Experiment 3 was conducted in the same manner as Experiment 2. The overall AUC shape and values (Fig. 5) more closely align with the results of Experiment 2 – low mismatch prevalence reduced discriminability under all three types of feedback. The area spanned by low mismatch prevalence (pAUC = .41) was less than that spanned by medium mismatch prevalence (pAUC = .45), *D* = 2.67, *p* = .007. For the comparison between low and high mismatch prevalence, the area spanned by low prevalence (pAUC = .34) was less than that spanned by high mismatch prevalence (pAUC = .39), *D* = 2.73, *p* = .006. Medium and high conditions were equivalent, *p* > .4.

## General discussion

The primary aims of the present set of studies were threefold: (1) to determine the effects that varying levels of mismatch prevalence have on sensitivity and confidence ratings in a simulated serial face-identity verification task; (2) to examine whether feedback (error-only or completely trial-by-trial) interacted with mismatch prevalence in ways applicable to real-world scenarios, and (3) to compare facial stimuli varying from highly controlled images frequently used in the literature featuring low within-person variability, and two novel sets of face-matching scenarios that capture a broader range of within-person variability. To these ends, Experiment 1 served as a replication of Papesh and Goldinger (2014), varying mismatch prevalence from low (20%), medium (50%), to high (80%). In addition, it utilized controlled facial stimuli from the Glasgow Unfamiliar Face Database used throughout face perception research. Further, participants received feedback after making errors. Experiments 2 and 3 replicated these mismatch prevalence conditions but used facial stimuli from the *Selfies for Science* database (Weatherford et al., 2016) that paired controlled images taken several months apart (Experiment 2) or paired controlled images with ambient images. In addition, Experiments 2 and 3 manipulated feedback between participants by providing no, error-only feedback, or full trial-by-trial feedback.

Experiment 1 found that low prevalence shifted criterion but did not substantially reduce empirical discriminability. Using different stimuli and varying feedback over a fuller range, Experiment 2 found that low mismatch prevalence both shifted criterion and reduced discriminability. Feedback (either trial by trial or only in the case of errors) did not improve performance. Experiment 3 broadened the range of within-person variability, having participants compare naturalistic ambient images to controlled images so that the matching task better corresponded to what identity screeners experience in real-world settings, with results similar to Experiment 2.

### Implications for the low-prevalence effect

Results from the current experiments lend support toward the criterion-shift explanation of the LPE (e.g., Wolfe et al., 2007), which proffers that, as mismatch prevalence decreases, so does the amount of information used to assert that two faces match.^4^ This, in turn, increases mismatch errors. Crossing our three levels of feedback with three levels of prevalence allowed us to observe whether participants shifted their response criteria across our three experiments. Our explanation of how feedback would manifest as shifting criterion was rooted in Kluger and DeNisi’s (1996) Feedback Intervention Theory. In the current experiments, we predicted that feedback would operate by shifting attention in presented face pairs toward cues that are typically most diagnostic to identity, and that feedback would interact with varying prevalence by offering fewer error signals to individuals in the low-prevalence condition than to those in medium and high-prevalence conditions. Such an explanation would imply a shift in criterion that is magnified by feedback. Our feedback manipulation was successful in affecting decision-making in a straightforward enough way to indicate a shift in criterion as we predicted. The smaller partial areas under the ROC curves observed in low-prevalence conditions across all three of the current experiments indicates that participants were making more mismatch errors under comparatively higher confidence when mismatch rates were low. As they were maximizing the scope of information available to make identity decisions, we interpret this as evidence that participants in low-prevalence conditions were utilizing a more liberal response criterion compared to participants in conditions with medium and high-prevalence conditions.

### Security applications and future directions

The current experiments also expand upon a growing multidisciplinary research literature aimed at aiding security and crime control concerns throughout the world. Our use of an ecologically valid facial database consisting of a wide range of inter- and intra-individual differences, and our design manipulating mismatch prevalence reproduces a clearer picture of real-world identity-screening conditions.

Regarding further application, much of the extant research involving human facial-verification screeners has focused on ways of improving their recruitment and training in professional settings. As mentioned in the introduction to Experiment 2, the benefits of feedback in long-term training is generally taken as a given, with all major training regimens implementing post-decision feedback in some way (Towler et al., 2019). It is worth noting, however, that some of these regimens have been developed and are implemented without a systematic empirical basis. The experiments reported here demonstrate that feedback can indeed help facial-matching abilities in certain circumstances (White, Kemp, et al., 2014), but not all. If, as argued by Papesh et al. (2018), feedback is necessary to produce the LPE, then future studies would need to examine whether more realistic feedback (e.g., a passenger’s ability to produce corroborating documentation when a screener suspects a mismatch) may provide a more optimistic outcome. Feedback may not be the most effective intervention, but what are possible additions or alternatives?

One possibility is for security agencies to recruit face super-recognizers, or individuals who very easily recognize unfamiliar faces after even brief viewings (Bruce, Bindemann, & Lander, 2018). Bate et al. (Frowd et al., 2019) examined the face-memory abilities of police officers already known to exhibit superior face matching. Officers underwent several unique face-processing tasks, revealing that different individuals excel at different types of face matching (i.e., quickly recognizing a face within a crowd, matching a studied face within a small set of nearly identical faces, etc.). In addition, some individuals excel at avoiding foils while others’ strengths lie in choosing the correct person. The challenges in recruiting such individuals include the fact that, although their accuracy may be many standard deviations above average, they are not necessarily aware of their superiority and tests designed to detect super-recognition have not been firmly established. It is worth pointing out that, within the studies reported here, individuals were actually excluded from some analyses for exhibiting perfect performance on match or mismatch trial types.

Given the small number of available face super-recognizers and the limitations of the tests designed to find them, another alternative is developing more finely tuned training regimens that can render unfamiliar face processing as accurate as familiar face processing. Burton, Kramer, Ritchie, and Jenkins (2016) assert that familiar face processing is a qualitatively different phenomenon than unfamiliar face processing. However, exposure to multiple unique instances of the same face under different pose, lighting, expression, and temporal variations can improve unfamiliar face identification (Menon, White, & Kemp, 2015b). Therefore, any newly developed training program should incorporate abstraction of familiarity from multiple instances of the same faces, particularly among individuals whom the screener may be challenged by e.g., other-race faces; (Susa et al., 2019).

The task of an ID screener extends beyond facial comparison. Sophisticated ID documents also contain watermarks, holographic imagery, specialized inks, and other security features that screeners must also verify. It is possible that many screeners focus their attention and time on these aspects of the task and give little attention to face verification given the wide within-person variability that they encounter. In other words, screeners may simply verify that the ID is a legitimate government-issued document and then assume that the person providing it is also the person in the photo. One last possibility to counter this problem focuses not on the screeners themselves but upon the ID documents. If documents featured multiple images of the same face taken under different conditions like those listed above, they may help screeners to make more accurate, higher-confidence identity judgments (e.g., White, Burton, et al., 2014). A future investigation of the LPE under more realistic conditions like those reported here might include multiple facial images against which an ID can be compared, and this could in turn greatly reduce or eliminate the LPE.

## Supplementary information

**Additional file 1.** Reaction Time Data

## Data Availability

The datasets used and/or analyzed during the current study are available from the corresponding author on reasonable request.

## Abbreviations

AUC: Area under the curve
GUFD: Glasgow Unfamiliar Face Database
LPE: Low-prevalence effect
pAUC: Partial area under the curve
ROC: Receiver operating characteristic

## Footnotes

1 In order to calculate similarity, Bruce et al., 1999 asked participants to sort candidate images from the database into piles of similar-looking individuals. These similarity values represent the frequency with which identities were sorted into the same pile.

2 Papesh and Goldinger (2014) argued that these penalties served the dual purpose of encouraging participants to stay invested in the task, as well as simulating the real-world consequences of the two different incorrect decisions.

3 Although participant-submitted images did undergo minor alterations (e.g., cropping to remove background details) in Photoshop CS7, we retained most of the variability as representative of these image types more generally. Predictably, participants did submit mostly flattering images. However, we did not accept any images that were changed to alter facial structure (e.g., camera filters) or other means to artificially alter perceptual information (e.g., teeth-whitening, eye-brightening).

4 We also recorded decision reaction time data as well, which revealed no evidence supporting the early search termination account of the low prevalence effect (e.g., Fleck & Mitroff, 2007). Analyses of these data are reported in the “Additional file 1”
