# Supplementary material for: You shall not pass: how facial variability and feedback affect the detection of low-prevalence fake IDs
Source: Cogn Res Princ Implic. 2020 Jan 28;5:3. doi: 10.1186/s41235-019-0204-1 (PMC6987271; doi:10.1186/s41235-019-0204-1)
Supplement: Supplementary file 1 — Additional file 1. Reaction Time Data [file 41235_2019_204_MOESM1_ESM.docx]

**Reaction Time Data**

**Experiment 1.** In all experiments, we trimmed reaction time (RT) data by establishing a cutoff score of 2.5 standard deviations above each participant’s mean trial RT with which to replace extreme values (Ratcliff, 1993). Of the original sample, we excluded data from *n*=4 participants (*n*=1 participant had more than 5% of trials in excess of this cutoff score; *n*=3 participants assigned to the high mismatch prevalence condition exhibited perfect match accuracy). We analyzed RTs using a 2 (Match Type-within: match, mismatch) X 3 (Mismatch prevalence-between: 80%, 50%, 20%) X 2 (Accuracy-within: correct, incorrect) mixed methods ANOVA. We found a main effect of match type, *F*(1,85) = 4.41, *p* = .033, *η^2^_p_* = .05, and no main effect of mismatch prevalence, *F*(2,85) = 1.80, *p* =.171, *η^2^_p_* = .04, and a main effect of accuracy, *F*(1,85) = 88.34, *p* < .001, *η^2^_p_* = .51. More importantly, these main effects were qualified by a three-way interaction, *F*(2,85) = 9.99, *p* < .001, *η^2^_p_* = .19. Although this three-way interaction was significant, longer reaction times were associated with poorer identification performance. Therefore, our data do not support that inaccurate trials, regardless of mismatch prevalence and match type, were the result of early search termination. However, errors did not appear to be attributable to early search termination — participants simply took longer before making any type of incorrect decisions and longer RTs was associated with *decreased* accuracy. Therefore, we do not find any support for an early search termination explanation of the LPE.

**Experiment 2.** Of the original sample, we excluded data from *n*=1 participant (assigned to the low prevalence condition who exhibited perfect mismatch accuracy). We found a significant interaction between match type, mismatch prevalence, and feedback for correct trials, *F*(1,74) = 4.63, *p* = .001, *η^2^_p_* = .20, and incorrect trials *F*(1,74) = 3.30, *p* = .015, *η^2^_p_* = .15. Again, however, early search termination did not produce a sufficient explanation for the accuracy differences. Instead, our evidence points to feedback generally increasing RT, but that additional time was insufficient to combat LPE errors.

Participants who took longer to make their decision were less likely to be correct. Extra consideration and decision time did not improve task performance. These data follow many other works dealing with the relationship between facial identification and decision processes. Unlike object processing, many studies in the eyewitness domain find that shorter reaction times are predictive of higher accuracy (e.g., Bornstein, Deffenbacher, Steven, & Mcgorty, 2012).

**Experiment 3.** Of the original sample, we excluded data from *n*=6 participants (*n*=3 participants had more than 5% of trials in excess of this cutoff score; *n*=3 participants assigned to the high prevalence condition exhibited perfect match accuracy). We found a significant interaction between match type, prevalence, and feedback for correct trials, *F*(1,68) = 3.01, *p* = .011, *η^2^_p_* = .210, and incorrect trials *F*(1,68) = 3.76, *p* = .003, *η^2^_p_* = .249. Again, however, early search termination did not produce a sufficient explanation for the accuracy differences. Instead, our evidence points to feedback generally increasing RT, but that additional time was not sufficient to combat LPE errors.

Overall, the early search termination account would have manifested as faster erroneous mismatch responses in low-prevalence conditions compared to high and medium prevalence conditions. We found no such evidence across the current experiments. In fact, only the longest reaction times reliably predicted errors. Of course, decision reaction time is only an approximation of search termination in a self-paced task such as ours, so early search termination cannot be completely disregarded by our results. Nevertheless, this pattern fits with the extant literature regarding the inverse relationship between decision time and facial identification accuracy. Future research will need to more strongly compare object and facial recognition paradigms to identify the shared and distinct mechanisms that may be guided by stimulus type.
